# Perceptions and Opinions of Patients About Mental Health Chatbots: Scoping Review

**DOI:** 10.2196/17828

**Published:** 2021-01-13

**Authors:** Alaa A Abd-Alrazaq, Mohannad Alajlani, Nashva Ali, Kerstin Denecke, Bridgette M Bewick, Mowafa Househ

**Affiliations:** 1 Division of Information and Computing Technology College of Science and Engineering, Hamad Bin Khalifa University Qatar Foundation Doha Qatar; 2 Institute of Digital Healthcare University of Warwick Warwick United Kingdom; 3 Institute for Medical Informatics Bern University of Applied Science Bern Switzerland; 4 Leeds Institute of Health Sciences School of Medicine University of Leeds Leeds United Kingdom

**Keywords:** chatbots, conversational agents, mental health, mental disorders, perceptions, opinions, mobile phone

## Abstract

**Background:**

Chatbots have been used in the last decade to improve access to mental health care services. Perceptions and opinions of patients influence the adoption of chatbots for health care. Many studies have been conducted to assess the perceptions and opinions of patients about mental health chatbots. To the best of our knowledge, there has been no review of the evidence surrounding perceptions and opinions of patients about mental health chatbots.

**Objective:**

This study aims to conduct a scoping review of the perceptions and opinions of patients about chatbots for mental health.

**Methods:**

The scoping review was carried out in line with the PRISMA (Preferred Reporting Items for Systematic reviews and Meta-Analyses) extension for scoping reviews guidelines. Studies were identified by searching 8 electronic databases (eg, MEDLINE and Embase) in addition to conducting backward and forward reference list checking of the included studies and relevant reviews. In total, 2 reviewers independently selected studies and extracted data from the included studies. Data were synthesized using thematic analysis.

**Results:**

Of 1072 citations retrieved, 37 unique studies were included in the review. The thematic analysis generated 10 themes from the findings of the studies: usefulness, ease of use, responsiveness, understandability, acceptability, attractiveness, trustworthiness, enjoyability, content, and comparisons.

**Conclusions:**

The results demonstrated overall positive perceptions and opinions of patients about chatbots for mental health. Important issues to be addressed in the future are the linguistic capabilities of the chatbots: they have to be able to deal adequately with unexpected user input, provide high-quality responses, and have to show high variability in responses. To be useful for clinical practice, we have to find ways to harmonize chatbot content with individual treatment recommendations, that is, a personalization of chatbot conversations is required.

## Introduction

### Background

Mental disorders are a growing global concern. Approximately 29% of individuals may experience such disorders in their lifetime [1]. Mental disorders have a negative effect on the quality of life and are one of the most common causes of disability [2]. Globally, the lost labor and capital output caused by mental disorders are predicted to cost US $16 trillion between 2011 and 2030 [3]. For many, accessing mental health care services is challenging because of the shortage of mental health care providers [4-7], lack of health care insurance coverage [8], and perceived stigma [9-11]. The lack of access to mental health care services increases the risk of self-harm and suicide [12,13].

Technological advancements have improved access to mental health care services [3]. According to the World Health Organization, more than one-fourth of 15,000 mobile health (mHealth) apps focus on mental health diagnosis or support [13]. Chatbots, also called conversational agents, virtual agents, and dialog systems, are one of the main mobile apps used for mental health.

Chatbots are programs able to converse and interact with a human using voice, text, and animation [14]. There are 2 types of chatbots: rule-based chatbots and intelligent chatbots. Although the former uses some predefined rules or decision trees to manage its response and dialog, the latter uses artificial intelligence (AI) to generate its dialog [14]. Chatbots have been employed in businesses across different industries, such as e-commerce and retail (eg, eBay’s ShopBot), hospitality (eg, Chatobook), real estate (eg, Apartment Ocean), entertainment (eg, Mojihunt), news (CNN’s Chatbot), travel (eg, Hello Hipmunk), finance and banking (eg, Wells Fargo’s chatbot), insurance (eg, ABIE), education (eg, Feed.Mind), legal (eg, Immigration Virtual Assistant), and personal assistance (eg, Apple’s Siri). In addition to the abovementioned industries, chatbots have become popular in health care (in general) and mental health (in specific) in the past 5 years. According to a scoping review conducted by Abd-alrazaq et al [14], there were 41 different chatbots used for several purposes in mental health, such as therapy, training, education, counseling, and screening. A systematic review of 12 studies showed that chatbots are effective in improving some mental disorders, such as depression, stress, and acrophobia [15].

### Research Problem and Aim

The adoption of new technology relies on the perceptions and opinions of users. Numerous studies have been conducted to assess the perceptions and opinions of patients about mental health chatbots [14]. There is a need to explore perceptions and opinions that may help in predicting the adoption of chatbots and improving them [14]. Although several reviews have been conducted on chatbots in mental health [4,14-17], none have summarized the findings of previous studies regarding perceptions and opinions of patients about mental health chatbots. Accordingly, the aim of this study is to review the perceptions and opinions of patients about mental health chatbots, as reported in the previous literature.

## Methods

### Study Design

We conducted a scoping review to accomplish this objective. A scoping review was conducted as the aim was to map the body of literature on this topic [18]. Owing to the broad nature of the inquiry, we expected a diversity of study designs. Scoping reviews are more suited to broader aims [18]. As we were not seeking to summarize the best available research on a specific question, a systematic review was not the ideal choice. Using chatbots for mental health is a field in relative infancy; therefore, a broader aim was necessary. The range of study designs currently used in the field makes equitable risk of bias assessment difficult; it is acknowledged that the risk of bias assessments is not required in scoping reviews [18,19]. Scoping reviews are generally accepted as more appropriate when diversity of study designs is expected. The PRISMA (Preferred Reporting Items for Systematic reviews and Meta-Analyses) Extension for Scoping Reviews guidelines were followed to carry out a systematic and transparent review [20].

### Search Strategy

#### Search Sources

The following electronic databases were searched in the current review: MEDLINE (via Ovid), Embase (via Ovid), PsycINFO (via Ovid), Scopus, Cochrane Central Register of Controlled Trials, IEEE Xplore, ACM Digital Library, and Google Scholar. Given that Google Scholar usually finds several thousands of references, which are ordered by their relevance to the search topic, we screened only the first 100 references [14,15,21]; these references are the most relevant. The search was conducted from October 25 to October 28, 2019. We also conducted backward reference list checking, where reference lists of the included studies and reviews on the search topic were screened for additional studies of relevance to the review. In addition, we carried out forward reference list checking, where the *cited by* function available in Google Scholar was used to find and screen studies that cited the included studies.

#### Search Terms

To derive search terms, we checked previous literature reviews [4,14-17] and opinions of informatics experts interested in mental health (which were collected in informal meetings). The search terms were selected based on the target intervention (eg, chatbots and conversational agents) and condition (eg, mental disorder and anxiety). The controlled vocabulary search terms (eg, MeSH, Emtree) were used, as appropriate. The search strings used for searching each electronic database are detailed in Multimedia Appendix 1.

### Study Eligibility Criteria

The intervention of interest in this review was chatbots that operate as stand-alone software or a web browser (Textboxes 1 and 2). We excluded chatbots that were integrated into robotics, serious games, SMS, or telephone systems and those that depended on human operator–generated dialog. No restrictions were applied regarding the type of dialog initiative (ie, use, system, mixed) and input and output modality (ie, spoken, visual, and written). The eligible population included individuals who used chatbots to improve their psychological well-being or mental disorders but not physicians or caregivers who use chatbots for their patients. To be included, studies had to assess patients’ perceptions and opinions about chatbots for mental health. The review included peer-reviewed articles, dissertations, and conference proceedings, and it excluded reviews, proposals, editorials, and conference abstracts. Only studies written in English were included in this review. No restrictions were applied regarding the study design, study setting, comparator, year of publication, or country of publication.

**Inclusion criteria.**

- Intervention: chatbots operate as stand-alone software or a web browser
- Population: patients who use chatbots for improving their psychological well-being or mental disorders
- Outcome: patients’ perceptions and opinions about mental health chatbots
- Type of publication: peer-reviewed articles, dissertations, and conference proceedings
- Language: English

**Exclusion criteria.**

- Intervention: chatbots integrated into robotics, serious games, SMS, or telephone systems and those depend on human operator–generated dialog
- Population: physicians or caregivers who use chatbots for improving their psychological well-being or mental disorders
- Outcome: other outcomes
- Type of publication: reviews, proposals, editorials, and conference abstracts
- Language: other languages

### Study Selection

In this review, MA and NA independently screened the titles and abstracts of all retrieved studies and independently read the full texts of studies included from the first step. AA resolved any disagreements between the reviewers. Cohen kappa was calculated to assess the intercoder agreement [22], which was 0.86 and 0.90 for screening titles and abstracts and for reading full texts, respectively, indicating excellent agreement [23].

### Data Extraction

Multimedia Appendix 2 shows the data extraction form used in this review, which was pilot tested using 4 included studies. Data were extracted from the included studies by 2 reviewers independently (MA and NA), and the third reviewer (AA) resolved any discrepancies in decisions between the reviewers. Cohen kappa showed an excellent agreement (0.83) [23].

### Assessment of Risk of Bias

Scoping reviews do not usually assess the risk of bias of the included studies because they have broad aims and include studies with diverse study designs [18,19]. The aim of this review was very broad, and the included studies had different study designs. Thus, the risk of bias of the included studies was not assessed in this review.

### Data Synthesis

A narrative approach was used to synthesize the data extracted from the included studies. Thematic analysis was used to generate themes based on the findings of the included studies. This data synthesis approach (ie, thematic analysis) has been applied in numerous systematic and scoping reviews [24-28]. Given the exploratory nature of this review, an inductive approach was used to generate themes directly from the data [29]. The thematic analysis was conducted following the steps proposed by Braun and Clarke [30], as it is the most systematic guide for conducting thematic analysis to date [31,32]. Specifically, the first author (AA) scrutinized and rescrutinized the extracted data to familiarize himself with it. AA then coded the data systematically. On the basis of the assigned codes, themes were generated. All authors checked the fit of the generated themes to the extracted data and assigned codes. Where authors had differing opinions on the assigned codes and/or generated themes, these discrepancies were resolved through discussion. Finally, all authors participated in defining and naming the themes. Microsoft Excel was used to manage the analysis process.

## Results

### Search Results

**Figure 1:**
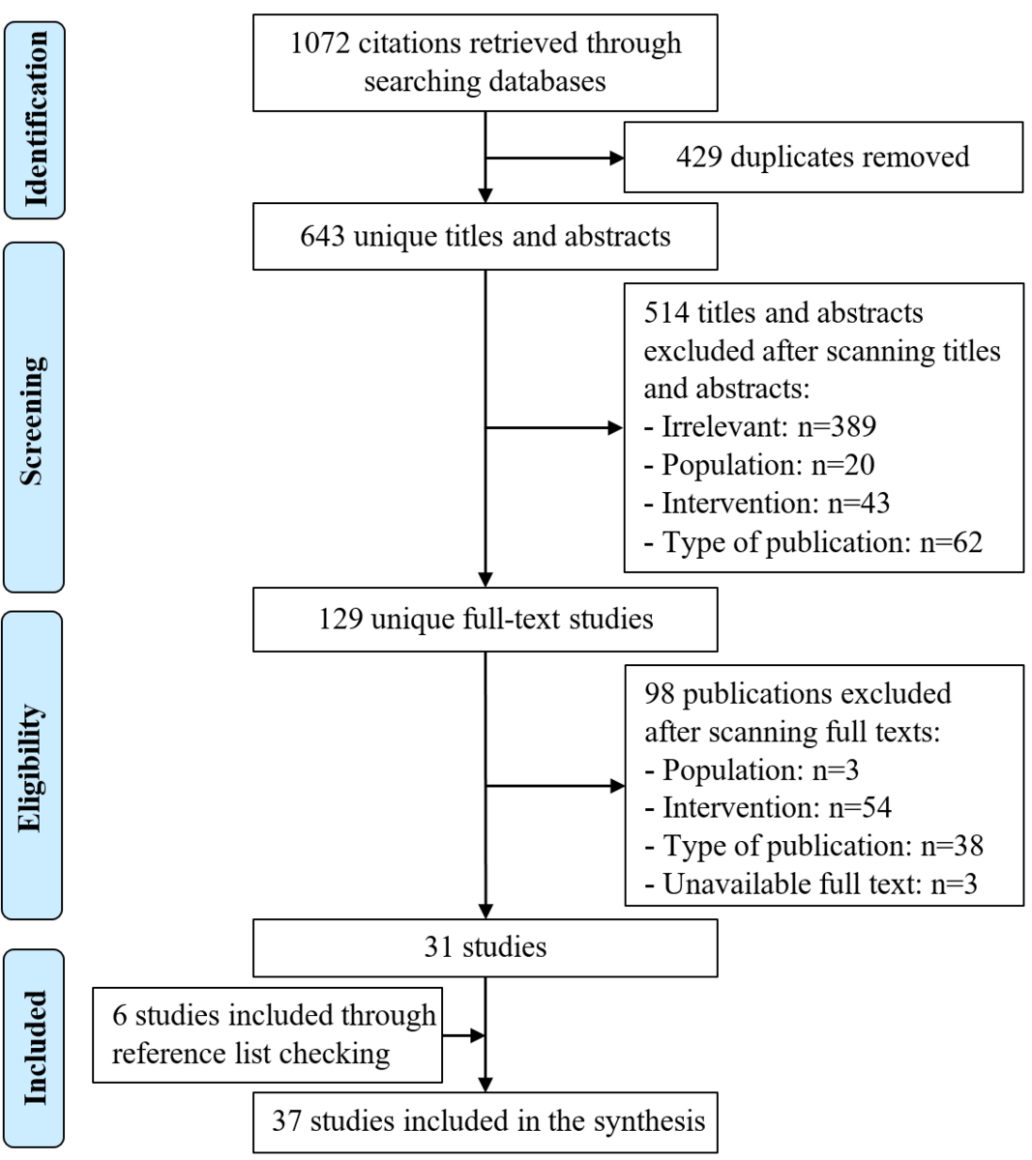
Flowchart of the study selection process.

As shown in Figure 1, 1072 citations were found by searching the electronic databases. After removing 429 duplicates of these citations, 643 titles and abstracts were screened. In the screening process, we excluded 514 titles and abstracts. Reading the full text of the remaining 129 citations resulted in a further 98 citations being excluded. The reasons for the exclusion are detailed in Figure 1. In backward and forward reference checking, we found 6 additional studies. In total, 37 studies were included in this review.

### Characteristics of Included Studies

As shown in Table 1, the most commonly used study design was a cross-sectional survey (34/37, 92%). About two-thirds of the studies were published as journal articles (24/37, 65%). The included studies were conducted in more than 15 countries, but approximately 46% (17/37) of them were carried out in the United States. Approximately 62% (23/37) of the studies were published between 2015 and 2019.

**Table 1:** Characteristics of the included studies.

Parameters and characteristics			Studies^a^	
**Study metadata, n (%)**				
	**Study design**			
		Survey		34 (92)
		Quasi-experiment		2 (5)
		Randomized controlled trial		1 (3)
	**Type of publication**			
		Journal article		24 (65)
		Conference proceeding		12 (32)
		Thesis		1 (3)
	**Country**			
		United States		17 (46)
		Australia		3 (8)
		France		3 (8)
		The Netherlands		3 (8)
		Japan		2 (5)
		Germany		1 (3)
		Korea		1 (3)
		Spain		1 (3)
		Sweden		1 (3)
		Turkey		1 (3)
		United Kingdom		1 (3)
		Romania, Spain, and Scotland		1 (3)
		Spain and Mexico		1 (3)
		Global population		1 (3)
	**Year of publication**			
		Before 2010		3 (8)
		2010-2014		11 (30)
		2015-2019		23 (62)
**Population** **characteristics**				
	**Sample size, n (%)**			
		≤50		24 (65)
		51-100		5 (14)
		101-200		6 (16)
		>200		2 (5)
	**Age (years)**			
		Mean (range)^b^		33.4 (13-79)
	**Sex** **, n (%)**			
		Male^c^		1436 (50)
	**Sample type, n (%)**			
		Clinical sample		21 (57)
		Nonclinical sample		16 (43)
	**Setting, n (%)^d^**			
		Clinical		14 (38)
		Educational		12 (32)
		Community		8 (22)
**Intervention characteristics, n (%)**				
	**Purpose**			
		Therapy		12 (32)
		Training		9 (24)
		Self-management		6 (16)
		Counseling		5 (14)
		Screening		4 (11)
		Diagnosing		1 (3)
	**Platform**			
		Stand-alone software		24 (65)
		Web based		13 (35)
	**Response generation**			
		Rule based		32 (86)
		Artificial intelligence		5 (14)
	**Dialog initiative**			
		Chatbot		32 (86)
		Both		5 (14)
	**Embodiment**			
		Yes		30 (81)
		No		7 (19)
	**Targeted disorders^e^**			
		Depression		41 (23)
		Autism		6 (16)
		Anxiety		6 (16)
		Any mental disorder		6 (16)
		Substance use disorder		5 (14)
		Posttraumatic stress disorder		5 (14)
		Schizophrenia		3 (8)
		Stress		3 (8)

^a^Percentages were rounded and may not sum to 100.

^b^Mean age was reported in 24 studies.

^c^Sex was reported in 29 studies.

^d^Setting was reported in 34 studies.

^e^Numbers do not add up as several chatbots target more than one health condition.

The sample size was 50 or less in 24 studies and more than 200 in 2 studies (Table 1). The participants’ age was reported in 24 studies, with a mean age of participants was 33.4 years (SD 15.2; range 13-79 years). The sex of participants was reported in 29 studies, where the mean percentage of men was 49.5%. In 57% (21/37) of the studies, participants were from clinical populations (ie, they had mental disorders). The sample was recruited from clinical (n=14), educational (n=12), or community settings (n=8). Multimedia Appendix 3 shows the metadata and population characteristics of each included study.

The 37 included studies assessed patients’ perceptions and opinions about 32 different chatbots. Chatbots were used for therapeutic purposes (n=12), training (n=9), self-management (n=6) counseling (n=5), screening (n=4), and diagnosis (n=1; Table 1). Chatbots were implemented in stand-alone software in 65% (24/37) of the studies, whereas the remaining chatbots were implemented in web-based platforms. In the majority of studies (32/37, 86.5%), chatbots generated their responses based on predefined rules or decision trees (rule based). Chatbots in the remaining studies generated their responses based on machine learning approaches. Chatbots led the dialog in most studies (n=32/37, 86.5%); both chatbots and users could lead the dialog in the remaining studies. Virtual agents (eg, avatar or virtual human) were embodied in chatbots in 30 of 37 studies (81.1%). The most common disorder targeted by chatbots was depression (n=15, 40.5%). Multimedia Appendix 4 shows the characteristics of the intervention in each included study [33-69].

### Study Findings

The thematic analysis generated 10 themes from the findings of the studies: usefulness, ease of use, responsiveness, understandability, acceptability, attractiveness, trustworthiness, enjoyability, content, and comparisons. More details about these themes are elaborated in the following subsections.

#### Theme 1: Usefulness

In total, 20 studies investigated the usefulness of chatbots and/or their features for patients [33-52]. In 3 studies [41,47-49,51], the overall usefulness of chatbots was rated as high. Participants reported that chatbots are useful for practicing conversations in a private place [33,46], learning [37,38,42,46], making users feel better [40], preparing users for interactions with health care providers [43], implementing the learned skills in daily life [46], facilitating a sense of accountability from daily check-in [37,38], keeping the learned skills more prominently in users’ minds [46], increasing users’ self-efficacy [46], improving users’ self-confidence and readiness for job interviews [47-49], and recalling users’ memories [51]. However, participants in one study doubted the usefulness of chatbots for smoking cessation [39].

Users considered the following components of chatbots useful: real-time feedback [33,45,50], diary [52], weekly summary [42], presenting the helpline during the conversation [36], and psychoeducation [52]. Some studies found that virtual agents embodied in chatbots are useful for motivating users to use chatbots [34], establishing a relationship with them [35], and feeling supported by them [45]. However, other studies demonstrated that participants had neutral perceptions and opinions about the added value of embodied virtual agents with speech [52] or without speech [44].

#### Theme 2: Ease of Use

The ease of use and usability of chatbots were assessed in 20 studies [33,34,36,39,43,46-51,53-61]. Participants in 15 studies rated the overall ease of use of chatbots as high [36,39,43,47-50,53-55,57-61]. A total of 5 studies assessed the usability of chatbots [34,36,46,51,56], and it was rated high in all these studies (ranging from 69 to 88.2). Participants in 3 studies reported that chatbots are easy to learn and become familiar with [33,39,55]. Although participants did not find chatbots difficult to navigate in one study [33], they rated the navigation of the chatbot as low in another study [36].

In 3 studies, participants faced difficulty in using the chatbot because they did not know when [60] and how [61] to reply to it, and they did not have enough options to reply to it [62]. Some participants in 3 studies criticized chatbots because of technical glitches that happened during their use, such as screen freezing [37,45,60]. Users suggested that chatbots allow them to respond by speaking and not typing to make them easy to use [57].

#### Theme 3: Responsiveness

This theme brings together perceptions and opinions of participants about verbal and nonverbal responses generated by chatbots in terms of realism, repetitiveness (variability), speed, friendliness, and empathy. A total of 10 studies assessed participants’ perceptions and opinions about how real the chatbots were in terms of verbal and nonverbal responses. Although participants in 7 studies had mixed or neutral perceptions and opinions about the realism of verbal and nonverbal responses [33,35,38,45,46,50,56], most participants in 3 studies held corresponding positive perceptions and opinions [52,57,60]. Participants believed that chatbots may be more realistic if they have an embodied virtual agent [44] and are able to communicate verbally [33].

Most participants in several studies stated that chatbots were able to show friendly [34,52,59,60,63] and emotional [35,37,38] responsiveness. However, participants in other studies had mixed perceptions and opinions about the ability of chatbots to generate friendly [35,44,64] and emotional [64] responses. Participants in one study found chatbots with an embodied virtual agent friendlier than those without an embodied virtual agent [44].

A total of 7 studies demonstrated that chatbot responses were repetitive [35-38,40,42,61]. Participants in one study reported that the repetitiveness of responses made the chatbot look less real [42]. Moreover, 3 studies concluded that the speed of chatbot responses was appropriate [57,60,61]. Yet, participants in 2 studies criticized the speed of chatbot responses as either too fast [38] or too slow [33]. In 6 studies, participants did not face any difficulties in understanding and interpreting chatbot responses [45,57,59,60,65,66].

In general, participants in 6 studies were satisfied with chatbot responses [33-36,62,63,67]. Participants attributed their satisfaction to the accuracy and consistency of chatbot feedback with what their therapist recommended in the past [33], appropriate use of high-quality elements (eg, Graphics Interchange Format images) [36], consistency of voice and tone of the chatbot [36], and quality of information provided [62,63]. However, participants in 4 studies were not satisfied by the conversation of chatbot because of confusing responses [57], disturbing users [40], the robotic quality of its voice [62], using emoticons (emojis) [37], conversations being too short [37], and shallowness of the conversations [42].

Participants suggested several enhancements related to the responsiveness of chatbots, such as the ability to speak [44], more flowing speech [33], friendlier voice [44], immediate responses [33,45], faster blinks [33], more explanation [33], providing feedback on each answered question [44], and more precise feedback [45].

#### Theme 4: Understandability

This theme brings together perceptions and opinions of participants about the ability of chatbots to understand their verbal and nonverbal contact. Chatbot understandability for verbal responses was rated as high among participants in 3 studies [33,45,61]; however, participants in other studies criticized the inability of the chatbot to understand their verbal responses in general [36-38,40,57], misspelled words (eg, anious instead of anxious) [36], different languages [36], unexpected answers [37,38], and nonverbal responses (eg, images, emojis, and facial expressions) [33,36,56]. Therefore, participants in 3 studies suggested that future chatbots should better understand and recognize their verbal and nonverbal responses [33,37,43].

#### Theme 5: Acceptability

This theme concerns participants’ acceptability of chatbots and its functionalities and their intentions to use them in the future. The acceptability of chatbots was rated high by users in 12 studies [34,37,38,43,45,46,53,54,57,61,66,68]. Wearing eye tracker goggles [62] or the inclusion of an embodied virtual agent [65] was acceptable for participants. There were mixed opinions about the acceptability of chatbots in one study [35]. Chatbots connected with a perception system (camera) for extracting data about user behavior was not acceptable for most participants in one study [60]. Users in one study indicated that the acceptability of chatbots could increase if the purpose of chatbots was clearly explained [33]. Note that the lack of clarity of the purpose of chatbots was highlighted by participants in 2 studies [33,42].

Furthermore, 6 studies demonstrated that people would like to use chatbots in the future [35,43,45,46,50,53,54,61,65], whereas participants in 2 studies were ambivalent about the future use of chatbots [33,39]. This ambivalence resulted from participants perceiving chatbots as a conversational tool for children [33]. Some participants reported that they probably would not use chatbots unless they popped up on their screens [33]. Although participants in one study showed high intention to use the chatbot in the future, they highlighted that it is inconvenient or inappropriate to use it every day [35].

#### Theme 6: Attractiveness

Participants in one study rated the attractiveness of a chatbot as low [57]. Furthermore, Demirci [55] found that perceptions and opinions of users about attractiveness changed from positive before using the chatbot to neutral after using it. Participants suggested improvements of the controls (eg, icons, combo boxes, buttons, and font size) [33,34] and the appearance of the embodied virtual agent [34] to obtain a more attractive graphical user interface.

#### Theme 7: Trustworthiness

This theme concerns participants’ trust in chatbot. In 7 studies, participants believed that chatbots are trustworthy [34,44,46,52,54,57,63]. One study concluded that participants were satisfied with the anonymity, confidentiality, and objectivity of chatbots [63]. Most participants in the 2 studies were comfortable to share and did share personal information with the chatbot [56,63].

#### Theme 8: Enjoyability

Participants in 9 studies considered using chatbots as enjoyable and fun [36,42-44,47-49,52,55,59]. However, one study found that perceptions and opinions of users about enjoyment changed from positive before using the chatbot to neutral after using it [55].

#### Theme 9: Content

This theme contains participants’ opinions about the content of chatbots. In 6 studies, participants were satisfied with the contents of chatbots such as videos, games, topics, suggestions, and weekly graphs [34,37,38,42,43,52]. However, the content of chatbots was criticized by users because of its superficiality [33,38], irrelevancy [38], having too long videos [37], and having overwhelming information [57]. Participants in 3 studies appreciated the personalization feature in chatbots that allowed them to customize the session length and the gender and appearance of the virtual agent [35,57,60]. Participants suggested that chatbots should contain psychoeducation [35], more therapy-based activities [34], longer and more frequent intervention sessions [43], more customizable content [35,43], and interventions for other chronic health conditions [43].

#### Theme 10: Comparisons

This theme brings together participant perspectives about chatbots in comparison with other chatbots or traditional methods. Although most participants in one study preferred interacting with a chatbot rather than a human for their health care [53], participants in another 2 studies had mixed preferences about that [33,45]. In 2 studies [44,58], participants preferred using chatbots with an embodied virtual agent compared with chatbots without an embodied virtual agent.

Participants in one study preferred that chatbot provides real-time feedback on their nonverbal behavior rather than postsession feedback [33]. According to Morris et al [67], participants preferred the chatbot’s responses drawn from an existing pool of web-based peer support data rather than those generated by the chatbot itself. Users preferred to use chatbots that provide information in an interactive fashion [43], are added on a device that they already own (eg, smartphones, tablets, computers) [60], and call them by their first name [60].

A chatbot without an embodied virtual agent (text-based chatbot) was compared with 2 chatbots with an embodied virtual agent (one reacts to the user with verbal and nonverbal empathic reactions, whereas the other did not) in another study [58]. The study found that the empathic chatbot was more acceptable, enjoyable, empathizing, understanding, nicer, sociable, trustworthy, realistic, private, anthropomorphic, animated, intelligent, socially influencing, friendlier, and safer than the nonempathic chatbot and the text-based chatbot [58]. Furthermore, the study demonstrated that participants are more willing to disclose information to the text-based chatbot than to empathic and nonempathic chatbots and a human counselor [58]. The study also found that participants were more willing to use empathic chatbots than nonempathic chatbots and text-based chatbots [58].

One study compared AI chatbots with an individual or a chatbot controlled by the same individual (Wizard-of-Oz) [56]. The study found that the Wizard-of-Oz chatbot was rated by participants as more usable and listened better than the AI chatbot [56]. Furthermore, users of the Wizard-of-Oz chatbot felt greater rapport than users of the AI chatbot and, surprisingly, than those who were interviewed by humans [56]. However, there was no difference between users of the AI chatbot and those interviewed by a human in their ratings of rapport [56].

In another study [69], participants felt a greater rapport with the real expert than with a rule-based chatbot. Participants also indicated that the rule-based chatbot is less able to understand their responses and feelings than a real expert [69]. Furthermore, participants found the real expert more trustworthy than the rule-based chatbot [69]. Participants reported that the chatbot is more enjoyable and engaging than traditional methods of learning and practicing dialectical behavior therapy skills [46].

## Discussion

### Principal Findings

The main finding of this review is that there are features of chatbots that health care providers cannot deliver over a long period. These features have been identified as useful in mental health chatbots: real-time feedback, weekly summary, and continuous data collection in terms of a diary. Usefulness and ease of use are aspects of chatbots that have been studied most comprehensively in the analyzed papers. Overall, the usefulness of mental health chatbots is perceived as high by patients. According to these studies, patients find chatbot systems easy to use. Interactional enjoyment and perceived trust are significant mediators of chatbot interaction [70]. Although chatbots are perceived as useful and easy to use, participants of reported studies recognized the existing conversational limitations of those systems: conversations are perceived as shallow, confusing, or too short. This points to an important issue to be addressed in future mental health chatbot development (see the *Practical and Research Implications* section). The conversation quality still needs to be improved. In this context, chatbot quality in terms of responsiveness and variability of responses is an important issue. Currently, systems are rather restricted in the number of responses, which might be because of the early development stage of many chatbots, as has already been reported by Laranjo et al [71]. Another relevant aspect judged important is the quality of provided information and consistency with recommendations of treating physicians. The implications of these principal findings toward practice and research are described in the *Practical and Research Implications* section.

### Comparison With Existing Literature

This is the first review that summarizes perceptions and opinions of patients about mental health chatbots, as reported by previous studies. Palanica et al [72] assessed physicians’ perceptions of health chatbots. They found that physicians see the benefits of those apps basically in support of patients in managing their health and improving physical, psychological, and behavioral health outcomes. As limitations of health chatbots, they mentioned the inability to comprehend and express human emotions and a lack of medical expert knowledge and intelligence. Our results provide the counterpart of this observation, namely, patients recognizing limitations in the conversation quality of health chatbots. A recent paper on a chatbot for regulating emotions shows that perceptions of patients and psychologists differ regarding attractiveness, efficiency, and stimulations of health chatbots [73]. Although psychologists see these aspects rather positive, patients are more critical and complain about the restrictions of chatbot conversations.

In their review of the landscape of psychiatric chatbots, Vaidyam [4] identified studies that showed high satisfaction with the interventions they received. Participants reported the interventions as helpful, easy to use, and informative and rated satisfaction highly (>4.2 out of 5) on all scales, including ease of use, desire to continue using the system, liking, and trust. In addition, the results of Bendig et al [16] suggest the practicability, feasibility, and acceptance of using chatbots to promote mental health. Our results confirm these observations: patients consider chatbots as useful, and acceptability is rated high.

A study assessed the use of mobile technologies in health-related areas from various perspectives [74]. It points to the following risks for mHealth app usage: lack of functionality, dissemination of false information, misdiagnosis, mistreatment, and unknown unwanted side effects. From the patient perspective, these issues could also be identified in our review: quality of provided information and consistency with recommendations of treating physicians are relevant aspects. The results of the study by Albrecht [74] go beyond our results by pointing to the risks of mHealth apps in case of an emergency. Implemented algorithms still lack reactivity (eg, in the case of self-endangerment or hazards of others). In addition, Singh et al [75] showed that only 23% of mHealth apps responded adequately to dangerous user input (eg, suicidal ideations). This illustrates the enormous need for improvement in terms of responsiveness of mHealth apps in potentially dangerous situations. According to our results, the patients did not seem to be too concerned about this issue of chatbot behavior in emergencies. It was not mentioned in the reviewed studies.

### Practical and Research Implications

#### Practical Implications

The study results have the following practical implications. To be useful, we need to create high-quality chatbots that are able to respond to a user in multiple ways. A mental health chatbot must be empathic to be perceived as motivating and engaging and to establish a relationship with the user. A study by de Gennaro [76] supports this by demonstrating that empathic chatbots have the potential to provide emotional support to victims of social exclusion.

The patient-doctor or patient-therapist relationship in standard health care settings is characterized by trust and loyalty. Measurements must be undertaken to make the chatbot-patient relationship also trustworthy. This could be realized by providing information on the secondary use of the collected patient data on data storage and analysis procedures. Another approach is blended therapy [77], a combination of face-to-face and web-based or digital therapy, which shows the potential of a cost-effective and accessible format in cognitive behavioral therapy. This would also address another practical implication, which is that the chatbot has to be related to the therapy. In particular, recommendations provided by a chatbot must be consistent with the recommendations of the treating health care professionals. This demands the integration of chatbots into the health care process; the chatbot should be aware of the recommendations or treatment plans of the health care provider. Finally, to increase the acceptance of chatbot use in patients, physicians need to be convinced of the usefulness of those systems so that they will recommend them to patients. Studies suggest that there are already physicians who are convinced of the usefulness [72]. Given the strong bond of trust among patients toward their physicians, patients will be convinced of the usefulness of an app once their physician recommends it.

#### Research Implications

From the practical implications, we can derive the following research implications. There is still a need to improve the linguistic capabilities of mental health chatbots [71]. Their ability to understand and react appropriately to user input has to be increased. Furthermore, methods are required to generate dynamic answers to ensure the variability of chatbot responses. Linguistic or lexical variability can be added to the knowledge base of rule-based chatbots, but the capabilities will always depend on the completeness of the knowledge base. Methods for slightly adapting or reformulating responses from the knowledge base could help in addressing this issue. In domains outside the health care domain, crowdsourcing has been applied to improve conversation quality [78]. However, in health care, we have to be careful with learning from data because we have to ensure that responses and recommendations are in line with clinical evidence. It is still an open research question on how to learn clinical evidence to train health chatbots.

Furthermore, methods have to be developed to deal with unexpected user input and to detect critical situations. In mental health, it is crucial to react appropriately for people who are at risk of suicide or self-harm [79]. Sentiment analysis methods have proven to be successful in analyzing social media messages with respect to suicide and self-harm [80]. These methods could be useful in health chatbots as well. The main challenge is the use of an appropriate reaction once an emergency situation has been detected. Another interesting research topic is the customization or personalization of chatbots to individual users. This topic is still in its infancy [81]. As long as mental health chatbots rely on decision trees or fix implemented rule bases, they will not be able to adapt to specific user needs. We can construct the knowledge base in such a way that there are responses for different types of users, but this will be time consuming and will always be incomplete. Learning from conversations with users could help. The style or complexity of language can be adapted based on the given user input. Patient-specific knowledge, for example, on treatment plans could be retrieved from health records. Methods are required to include such knowledge dynamically to a chatbot. In this way, the chatbot content is adapted to match individual needs.

For evaluating the mental health chatbot, benchmarks have to be created, and consistent metrics and methods have to be developed. Laranjo et al [71] reviewed the characteristics, current applications, and evaluation measures of health chatbots. Evaluation measures were divided into 3 main types: technical performance, user experience, and health research measures. The first attempts toward evaluation frameworks for digital health interventions [82] and health chatbots [83,84] have been recently published. Depending on the facet under consideration, different metrics can be used: system performance and effectiveness can be evaluated using different computational metrics (eg, usability, ease of use, usefulness) [85]. Software quality can be measured by reliability, security, maintainability, and efficiency using software engineering metrics [86]. If the system uses AI and machine learning techniques, the metrics comprise the accuracy and precision of predictions and recommendations. Furthermore, the efficiency of the systems has to be evaluated and compared with existing care models. With regard to safe app use, 3 criteria should be evaluated: (1) quality of the therapeutic content, (2) functionality, and (3) data safety and protection [87].

### Strengths and Limitations

#### Strengths

This review was developed, executed, and reported according to the PRISMA Extension for Scoping Reviews [20]. This enabled us to produce a high-quality review.

The most commonly used databases in health and information technology were searched to retrieved relevant studies as many as possible. Searching Google Scholar and carrying out backward and forward reference list checking enabled us to identify gray literature and minimize the risk of publication bias as much as possible. As no restrictions were applied regarding the study design, study setting, comparator, year of publication, and country of publication, this review can be considered comprehensive.

Selection bias in this review was minimal because study selection and data extraction were performed independently by 2 reviewers. Furthermore, the agreement between reviewers was very good for study selection and data extraction. This study is one of the few reviews that used thematic analysis to synthesize the findings of the included studies. The thematic analysis followed the highly recommended guidelines proposed by Braun and Clarke [30].

#### Limitations

This review focused on chatbots that only work on stand-alone software and a web browser (but not robotics, serious games, SMS, or telephones). Furthermore, this review was restricted to chatbots that are not controlled by human operators (Wizard-of-Oz). Therefore, perceptions and opinions of patients found in this review may be different from their perceptions and opinions about Wizard-of-Oz chatbots and/or chatbots with alternative modes of delivery. The abovementioned restrictions were applied by previous reviews about chatbots, as these features are not part of ordinary chatbots [4,14,17].

Owing to practical constraints, we restricted the search to English studies and we could not search interdisciplinary databases (eg, Web of Science and ProQuest), conduct manual search, or contact experts. Consequently, it is likely that we have missed some English and non-English studies. Most included studies were conducted in developed countries, particularly in the United States. Therefore, the findings of this review may not be generalizable to developing countries, as patients in such countries may have different perceptions and opinions about mental health chatbots.

### Conclusions

In this paper, we explored perceptions and opinions of patients about mental health chatbots, as reported in the existing literature. The results demonstrated that there are overall positive perceptions and opinions of patients about mental health chatbots, although there is some skepticism toward trustworthiness and usefulness. Many important aspects have been identified to be addressed in research and practice. Among them are the need to improve the linguistic capabilities of chatbots and seamless integration into the health care process. Future research will have to pick up those issues to create successful, well-perceived chatbot systems, and we will start developing corresponding concepts and methods. The research implications are also relevant for health care chatbots beyond mental health chatbots. Their consideration has the potential to improve patients’ perceptions of health care chatbots in general.

## Appendix

Multimedia Appendix 1 Search strategy.

Multimedia Appendix 2 Data extraction form.

Multimedia Appendix 3 The metadata and population characteristics of each included study.

Multimedia Appendix 4 Characteristics of the intervention in each included study.

## Abbreviations

AI: artificial intelligence
mHealth: mobile health
PRISMA: Preferred Reporting Items for Systematic reviews and Meta-Analyses
