# Supplementary material for: Perceptions and Opinions of Patients About Mental Health Chatbots: Scoping Review
Source: J Med Internet Res. 2021 Jan 13;23(1):e17828. doi: 10.2196/17828 (PMC7840290; doi:10.2196/17828)
Supplement: Multimedia Appendix 1 [file jmir_v23i1e17828_app1.docx]

Database(s): **PsycINFO**2002 to April Week 5 2019 
Search Strategy:

| **#** | **Searches** | **Results** |
| --- | --- | --- |
| 1 | exp Mental Disorders/ | 374453 |
| 2 | mental disorder*.tw. | 30822 |
| 3 | exp Mental Health/ | 49523 |
| 4 | Mental health.tw. | 129321 |
| 5 | exp Affective Disorders/ | 107823 |
| 6 | affective disorder*.tw. | 7988 |
| 7 | mood disorder*.tw. | 13365 |
| 8 | exp Psychosis/ | 62321 |
| 9 | psychosis.tw. | 25372 |
| 10 | psychotic disorder*.tw. | 7495 |
| 11 | exp Obsessive Compulsive Disorder/ | 9265 |
| 12 | obsessive-compulsive disorder*.tw. | 11310 |
| 13 | exp Autism Spectrum Disorders/ | 32780 |
| 14 | autism.tw. | 37121 |
| 15 | exp Panic Disorder/ | 3385 |
| 16 | panic disorder*.tw. | 5755 |
| 17 | exp Phobias/ | 6088 |
| 18 | phobia.tw. | 6019 |
| 19 | phobic disorder*.tw. | 189 |
| 20 | exp Posttraumatic Stress Disorder/ | 24321 |
| 21 | post-traumatic stress disorder*.tw. | 8352 |
| 22 | exp Drug Abuse/ | 64442 |
| 23 | drug abuse.tw. | 9680 |
| 24 | substance abuse.tw. | 23510 |
| 25 | drug dependence.tw. | 2353 |
| 26 | substance dependence.tw. | 2099 |
| 27 | exp ADDICTION/ | 30646 |
| 28 | addiction.tw. | 30565 |
| 29 | depression.tw. | 162153 |
| 30 | Depressed.tw. | 25159 |
| 31 | melancholia.tw. | 980 |
| 32 | exp Anxiety Disorders/ | 54165 |
| 33 | Anxiety disorder*.tw. | 26300 |
| 34 | anxious.tw. | 12720 |
| 35 | exp Bipolar Disorder/ | 18989 |
| 36 | bipolar.tw. | 28677 |
| 37 | schizophreni*.tw. | 65039 |
| 38 | exp SCHIZOPHRENIA/ | 47785 |
| 39 | exp Eating Disorders/ | 18278 |
| 40 | eating disorder*.tw. | 17888 |
| 41 | anorexia.tw. | 8823 |
| 42 | bulimia.tw. | 5360 |
| 43 | binge-eating.tw. | 4653 |
| 44 | conversational agent*.tw. | 185 |
| 45 | conversational bot*.tw. | 5 |
| 46 | conversational system*.tw. | 16 |
| 47 | conversational interface*.tw. | 7 |
| 48 | chatbot*.tw. | 49 |
| 49 | chat bot*.tw. | 13 |
| 50 | chat-bot*.tw. | 13 |
| 51 | chatter bot*.tw. | 1 |
| 52 | chatterbot*.tw. | 6 |
| 53 | smart bot*.tw. | 1 |
| 54 | smartbot*.tw. | 1 |
| 55 | smart-bot*.tw. | 1 |
| 56 | virtual agent*.tw. | 176 |
| 57 | virtual character*.tw. | 176 |
| 58 | virtual coach*.tw. | 38 |
| 59 | virtual human.tw. | 151 |
| 60 | avatar.tw. | 996 |
| 61 | embodied agent*.tw. | 129 |
| 62 | relational agent*.tw. | 23 |
| 63 | animated character*.tw. | 66 |
| 64 | 1 or 2 or 3 or 4 or 5 or 6 or 7 or 8 or 9 or 10 or 11 or 12 or 13 or 14 or 15 or 16 or 17 or 18 or 19 or 20 or 21 or 22 or 23 or 24 or 25 or 26 or 27 or 28 or 29 or 30 or 31 or 32 or 33 or 34 or 35 or 36 or 37 or 38 or 39 or 40 or 41 or 42 or 43 | 624325 |
| 65 | 44 or 45 or 46 or 47 or 48 or 49 or 50 or 51 or 52 or 53 or 54 or 55 or 56 or 57 or 58 or 59 or 60 or 61 or 62 or 63 | 1889 |
| 66 | 64 and 65 | 223 |

Database(s): **Ovid MEDLINE(R)**1996 to April Week 4 2019 
Search Strategy:

| **#** | **Searches** | **Results** |
| --- | --- | --- |
| 1 | exp Mental Disorders/ | 766580 |
| 2 | mental disorder*.tw. | 24765 |
| 3 | exp Mental Health/ | 25942 |
| 4 | Mental health.tw. | 87385 |
| 5 | exp Mood Disorders/ | 84443 |
| 6 | mood disorder*.tw. | 12481 |
| 7 | exp Psychotic Disorders/ | 24515 |
| 8 | psychotic disorder*.tw. | 6071 |
| 9 | exp Obsessive-Compulsive Disorder/ | 9561 |
| 10 | obsessive-compulsive disorder*.tw. | 9049 |
| 11 | exp Autism Spectrum Disorder/ | 21772 |
| 12 | autism.tw. | 28072 |
| 13 | exp Panic Disorder/ | 5512 |
| 14 | panic disorder*.tw. | 6123 |
| 15 | exp Phobic Disorders/ | 6346 |
| 16 | phobic disorder*.tw. | 212 |
| 17 | exp Stress Disorders, Post-Traumatic/ | 26901 |
| 18 | post-traumatic stress disorder*.tw. | 7380 |
| 19 | exp Substance-Related Disorders/ | 157981 |
| 20 | substance-related disorder*.tw. | 432 |
| 21 | exp Depression/ | 82931 |
| 22 | depression.tw. | 204340 |
| 23 | Depressed.tw. | 46470 |
| 24 | melancholia.tw. | 573 |
| 25 | exp Anxiety Disorders/ | 42793 |
| 26 | Anxiety disorder*.tw. | 21575 |
| 27 | anxious.tw. | 10836 |
| 28 | phobia.tw. | 4915 |
| 29 | exp Bipolar Disorder/ | 25670 |
| 30 | bipolar.tw. | 41045 |
| 31 | exp Schizophrenia/ | 60531 |
| 32 | schizophreni*.tw. | 71899 |
| 33 | psychosis.tw. | 21684 |
| 34 | exp "Feeding and Eating Disorders"/ | 19466 |
| 35 | eating disorder*.tw. | 13582 |
| 36 | anorexia.tw. | 17140 |
| 37 | binge-eating.tw. | 4086 |
| 38 | bulimia.tw. | 4679 |
| 39 | drug dependence.tw. | 2339 |
| 40 | substance dependence.tw. | 1718 |
| 41 | addiction.tw. | 27730 |
| 42 | drug abuse.tw. | 9279 |
| 43 | substance abuse.tw. | 168960 |
| 44 | conversational agent*.tw. | 46 |
| 45 | conversational bot*.tw. | 1 |
| 46 | conversational system*.tw. | 4 |
| 47 | conversational interface*.tw. | 3 |
| 48 | chatbot*.tw. | 11 |
| 49 | chat bot*.tw. | 11 |
| 50 | chatterbot*.tw. | 0 |
| 51 | chatter bot*.tw. | 0 |
| 52 | chat-bot*.tw. | 11 |
| 53 | smartbot*.tw. | 1 |
| 54 | smart bot*.tw. | 3 |
| 55 | smart-bot*.tw. | 3 |
| 56 | virtual coach*.tw. | 22 |
| 57 | virtual agent*.tw. | 43 |
| 58 | embodied agent*.tw. | 29 |
| 59 | relational agent*.tw. | 3 |
| 60 | avatar.tw. | 492 |
| 61 | virtual character*.tw. | 90 |
| 62 | animated character*.tw. | 31 |
| 63 | virtual human.tw. | 169 |
| 64 | 1 or 2 or 3 or 4 or 5 or 6 or 7 or 8 or 9 or 10 or 11 or 12 or 13 or 14 or 15 or 16 or 17 or 18 or 19 or 20 or 21 or 22 or 23 or 24 or 25 or 26 or 27 or 28 or 29 or 30 or 31 or 32 or 33 or 34 or 35 or 36 or 37 or 38 or 39 or 40 or 41 or 42 or 43 | 1018621 |
| 65 | 44 or 45 or 46 or 47 or 48 or 49 or 50 or 51 or 52 or 53 or 54 or 55 or 56 or 57 or 58 or 59 or 60 or 61 or 62 or 63 | 906 |
| 66 | 64 and 65 | 135 |

Database(s): **Embase**1996 to 2019 Week 18 
Search Strategy:

| **#** | **Searches** | **Results** |
| --- | --- | --- |
| 1 | exp mental disease/ | 1644370 |
| 2 | mental disorder*.tw. | 39872 |
| 3 | exp mental health/ | 127283 |
| 4 | Mental health.tw. | 141777 |
| 5 | exp mood disorder/ | 409105 |
| 6 | mood disorder*.tw. | 22775 |
| 7 | affective disorder*.tw. | 15162 |
| 8 | exp psychosis/ | 211473 |
| 9 | psychosis.tw. | 43163 |
| 10 | psychotic disorder*.tw. | 12264 |
| 11 | exp obsessive compulsive disorder/ | 32109 |
| 12 | obsessive-compulsive disorder*.tw. | 15080 |
| 13 | exp autism/ | 58150 |
| 14 | autism.tw. | 46816 |
| 15 | exp panic/ | 17912 |
| 16 | panic disorder*.tw. | 9170 |
| 17 | exp phobia/ | 24044 |
| 18 | phobia.tw. | 7879 |
| 19 | phobic disorder*.tw. | 305 |
| 20 | exp posttraumatic stress disorder/ | 51080 |
| 21 | post-traumatic stress disorder*.tw. | 12310 |
| 22 | exp drug dependence/ | 161320 |
| 23 | drug dependence.tw. | 3640 |
| 24 | drug abuse.tw. | 15432 |
| 25 | substance abuse.tw. | 26417 |
| 26 | substance dependence.tw. | 2672 |
| 27 | exp addiction/ | 204593 |
| 28 | addiction.tw. | 50599 |
| 29 | exp depression/ | 375981 |
| 30 | Depressed.tw. | 71076 |
| 31 | melancholia.tw. | 829 |
| 32 | exp anxiety disorder/ | 199776 |
| 33 | Anxiety disorder*.tw. | 36119 |
| 34 | anxious.tw. | 18323 |
| 35 | exp bipolar disorder/ | 55565 |
| 36 | bipolar.tw. | 73858 |
| 37 | exp schizophrenia/ | 137223 |
| 38 | schizophreni*.tw. | 122933 |
| 39 | exp eating disorder/ | 38745 |
| 40 | eating disorder*.tw. | 21316 |
| 41 | anorexia.tw. | 29772 |
| 42 | bulimia.tw. | 6786 |
| 43 | binge-eating.tw. | 6126 |
| 44 | conversational agent*.tw. | 79 |
| 45 | conversational bot*.tw. | 1 |
| 46 | conversational system*.tw. | 10 |
| 47 | conversational interface*.tw. | 4 |
| 48 | chat bot*.tw. | 22 |
| 49 | chatbot*.tw. | 51 |
| 50 | chat-bot*.tw. | 22 |
| 51 | chatterbot*.tw. | 0 |
| 52 | chatter bot*.tw. | 0 |
| 53 | smart bot*.tw. | 8 |
| 54 | smartbot*.tw. | 2 |
| 55 | smart-bot*.tw. | 7 |
| 56 | virtual agent*.tw. | 85 |
| 57 | virtual character*.tw. | 141 |
| 58 | virtual coach*.tw. | 43 |
| 59 | virtual human.tw. | 293 |
| 60 | avatar.tw. | 989 |
| 61 | embodied agent*.tw. | 41 |
| 62 | relational agent*.tw. | 6 |
| 63 | animated character*.tw. | 46 |
| 64 | 1 or 2 or 3 or 4 or 5 or 6 or 7 or 8 or 9 or 10 or 11 or 12 or 13 or 14 or 15 or 16 or 17 or 18 or 19 or 20 or 21 or 22 or 23 or 24 or 25 or 26 or 27 or 28 or 29 or 30 or 31 or 32 or 33 or 34 or 35 or 36 or 37 or 38 or 39 or 40 or 41 or 42 or 43 | 1854812 |
| 65 | 44 or 45 or 46 or 47 or 48 or 49 or 50 or 51 or 52 or 53 or 54 or 55 or 56 or 57 or 58 or 59 or 60 or 61 or 62 or 63 | 1705 |
| 66 | 64 and 65 | 356 |
| 67 | limit 66 to exclude medline journals | 91 |

Database(s): **IEEE Xplore**
Search Strategy:

| **#** | **Search results** | **Hit** |
| --- | --- | --- |
| 1 | (((((("Abstract":"Chat bot*") OR "Abstract":chatbot*) OR "Abstract":"Conversational agent*") OR "Abstract":avatar) OR "Abstract":"virtual agent*") AND "Abstract":mental) | 47 |
| 2 | (((((("Abstract":"Chat bot*") OR "Abstract":chatbot*) OR "Abstract":"Conversational agent*") OR "Abstract":avatar) OR "Abstract":"virtual agent*") AND "Abstract":mood) | 19 |
| 3 | (((((("Abstract":"Chat bot*") OR "Abstract":chatbot*) OR "Abstract":"Conversational agent*") OR "Abstract":avatar) OR "Abstract":"virtual agent*") AND "Abstract":depression) | 11 |
| 4 | (((((("Abstract":"Chat bot*") OR "Abstract":chatbot*) OR "Abstract":"Conversational agent*") OR "Abstract":avatar) OR "Abstract":"virtual agent*") AND "Abstract":anxiety) | 6 |
| 5 | (((((("Abstract":"Chat bot*") OR "Abstract":chatbot*) OR "Abstract":"Conversational agent*") OR "Abstract":avatar) OR "Abstract":"virtual agent*") AND "Abstract":autism) | 29 |
| 6 | (((((("Abstract":"Chat bot*") OR "Abstract":chatbot*) OR "Abstract":"Conversational agent*") OR "Abstract":avatar) OR "Abstract":"virtual agent*") AND "Abstract":panic) | 1 |
| 7 | (((((("Abstract":"Chat bot*") OR "Abstract":chatbot*) OR "Abstract":"Conversational agent*") OR "Abstract":avatar) OR "Abstract":"virtual agent*") AND "Abstract":phobia) | 7 |
| 8 | (((((("Abstract":"Chat bot*") OR "Abstract":chatbot*) OR "Abstract":"Conversational agent*") OR "Abstract":avatar) OR "Abstract":"virtual agent*") AND "Abstract":bipoler) | 0 |
| 9 | (((((("Abstract":"Chat bot*") OR "Abstract":chatbot*) OR "Abstract":"Conversational agent*") OR "Abstract":avatar) OR "Abstract":"virtual agent*") AND "Abstract”:schizophrenia) | 2 |
| 10 | (((((("Abstract":"Chat bot*") OR "Abstract":chatbot*) OR "Abstract":"Conversational agent*") OR "Abstract":avatar) OR "Abstract":"virtual agent*") AND "Abstract":post-traumatic stress) | 3 |
| 11 | (((((("Abstract":"Chat bot*") OR "Abstract":chatbot*) OR "Abstract":"Conversational agent*") OR "Abstract":avatar) OR "Abstract":"virtual agent*") AND "Abstract":eating disorder) | 0 |
| 12 | (((((("Abstract":"Chat bot*") OR "Abstract":chatbot*) OR "Abstract":"Conversational agent*") OR "Abstract":avatar) OR "Abstract":"virtual agent*") AND "Abstract":psychosis) | 0 |
| 13 | (((((("Abstract":"Chat bot*") OR "Abstract":chatbot*) OR "Abstract":"Conversational agent*") OR "Abstract":avatar) OR "Abstract":"virtual agent*") AND "Abstract":anorexia) | 0 |
| 14 | (((((("Abstract":"Chat bot*") OR "Abstract":chatbot*) OR "Abstract":"Conversational agent*") OR "Abstract":avatar) OR "Abstract":"virtual agent*") AND "Abstract":bulimia) | 0 |
| 15 | (((((("Abstract":"Chat bot*") OR "Abstract":chatbot*) OR "Abstract":"Conversational agent*") OR "Abstract":avatar) OR "Abstract":"virtual agent*") AND "Abstract":addiction) | 1 |
| 16 | (((((("Abstract":"Chat bot*") OR "Abstract":chatbot*) OR "Abstract":"Conversational agent*") OR "Abstract":avatar) OR "Abstract":"virtual agent*") AND "Abstract":bulimia) | 0 |
| 17 | (((((("Abstract":"Chat bot*") OR "Abstract":chatbot*) OR "Abstract":"Conversational agent*") OR "Abstract":avatar) OR "Abstract":"virtual agent*") AND "Abstract":drug dependence) | 0 |
| 18 | (((((("Abstract":"Chat bot*") OR "Abstract":chatbot*) OR "Abstract":"Conversational agent*") OR "Abstract":avatar) OR "Abstract":"virtual agent*") AND "Abstract":substance dependence) | 0 |
| 19 | (((((("Abstract":"Chat bot*") OR "Abstract":chatbot*) OR "Abstract":"Conversational agent*") OR "Abstract":avatar) OR "Abstract":"virtual agent*") AND "Abstract":drug abuse) | 0 |
| 20 | (((((("Abstract":"Chat bot*") OR "Abstract":chatbot*) OR "Abstract":"Conversational agent*") OR "Abstract":avatar) OR "Abstract":"virtual agent*") AND "Abstract":substance abuse) | 0 |
| **Total** | | 119 |

| **Databases** | **Search strings** | **Hit** |
| --- | --- | --- |
| ACM digital Library | ("mental disorder*" OR "mental health" OR "mood disorder*" OR "psychotic disorder*" OR "obsessive-compulsive disorder*" OR autism OR "panic disorder*" OR "phobic disorder*" OR "post-traumatic stress disorder*" OR "substance-related disorder*" OR depression OR depressed OR melancholia OR "anxiety disorder*" OR anxious OR phobia OR bipolar OR schizophrenia OR psychosis OR eating disorder* OR anorexia OR binge-eating OR bulimia OR "drug dependence" OR "substance dependence" OR addiction OR "drug abuse" OR "substance abuse") AND ("conversational agent*" OR "conversational bot*" OR "conversational system*" OR "conversational interface*" OR "chatbot*" OR "chat bot*" OR "chatterbot*" OR "chatter bot*" OR "chat-bot*" OR "smartbot*" OR "smart bot*" OR "smart-bot*" OR "virtual coach*" OR "virtual agent*" OR "embodied agent*" OR "relational agent*" OR avatar OR "virtual character*" OR "animated character*" OR "virtual human") | 69 |
| Cochrane central register for controlled trials | (("mental disorder*" OR "mental health" OR "mood disorder*" OR "psychotic disorder*" OR "obsessive-compulsive disorder*" OR autism OR "panic disorder*" OR "phobic disorder*" OR "post-traumatic stress disorder*" OR "substance-related disorder*" OR depression OR depressed OR melancholia OR "anxiety disorder*" OR anxious OR phobia OR bipolar OR schizophrenia OR psychosis OR eating disorder* OR anorexia OR binge-eating OR bulimia OR "drug dependence" OR "substance dependence" OR addiction OR "drug abuse" OR "substance abuse") AND ("conversational agent*" OR "conversational bot*" OR "conversational system*" OR "conversational interface*" OR "chatbot*" OR "chat bot*" OR "chatterbot*" OR "chatter bot*" OR "chat-bot*" OR "smartbot*" OR "smart bot*" OR "smart-bot*" OR "virtual coach*" OR "virtual agent*" OR "embodied agent*" OR "relational agent*" OR avatar OR "virtual character*" OR "animated character*" OR "virtual human")):ti,ab,kw (Word variations have been searched) | 82 |
| Scopus | TITLE-ABS-KEY ( "mental disorder*" OR "mental health" OR "mood disorder*" OR "psychotic disorder*" OR "obsessive-compulsive disorder*" OR autism OR "panic disorder*" OR "phobic disorder*" OR "post-traumatic stress disorder*" OR "substance-related disorder*" OR depression OR depressed OR melancholia OR "anxiety disorder*" OR anxious OR phobia OR bipolar OR schizophrenia OR psychosis OR eating AND disorder* OR anorexia OR binge-eating OR bulimia OR "drug dependence" OR "substance dependence" OR addiction OR "drug abuse" OR "substance abuse" ) AND TITLE-ABS-KEY ( "conversational agent*" OR "conversational bot*" OR "conversational system*" OR "conversational interface*" OR "chatbot*" OR "chat bot*" OR "chatterbot*" OR "chatter bot*" OR "chat-bot*" OR "smartbot*" OR "smart bot*" OR "smart-bot*" OR "virtual coach*" OR "virtual agent*" OR "embodied agent*" OR "relational agent*" OR avatar OR "virtual character*" OR "animated character*" OR "virtual human" ) AND ( LIMIT-TO ( SRCTYPE , "j" ) OR LIMIT-TO ( SRCTYPE , "p" ) ) | 253 |
| Google Scholar | ("mental disorder*" OR "mental health" OR "mood disorder*" OR autism OR depression OR anxiety OR phobia OR bipolar OR schizophrenia) AND ("conversational agent*" OR "chatbot*" OR "chat bot*" OR "chatterbot*") | 100 |
