# Supplementary material for: Perceptions and Opinions of Patients About Mental Health Chatbots: Scoping Review
Source: J Med Internet Res. 2021 Jan 13;23(1):e17828. doi: 10.2196/17828 (PMC7840290; doi:10.2196/17828)
Supplement: Multimedia Appendix 2 [file jmir_v23i1e17828_app2.docx]

**Multimedia Appendix 2**. Data extraction form.

| **Concept** | **Definition** |
| --- | --- |
| **Study Characteristics** |  |
| Author | The first author of the study. |
| Year of Publication | The year in which the study was published. |
| Country of publication | The country where the study was published. |
| Type of publication | The medium in which the study was published (e.g. conference proceedings, journal, thesis). |
| Study design | The research method that the study used to collect the data. |
| Study aim | What the study aimed to find out. |
| **Population characteristics** |  |
| Number of participants | Number of people who participated in the study. |
| Mean age | The average age of participants. |
| Gender (male) | Percentage of males in the sample. |
| Health condition | Health status of participants (e.g. clinical or non-clinical sample). |
| Recruitment Setting | Place where participants were recruited (i.e. clinical, educational, community settings). |
| **Intervention characteristics** |  |
| Chatbot name | Name of the chatbot. |
| Chatbot aim | What it is that the chatbot aims to achieve (therapy, education, counselling, self-management, screening, diagnosing) |
| Platform | The platform in which the chatbot was implemented (i.e. stand-alone software, web-based, or multimodal platform). |
| Response generation | The method of processing inputs and generating responses:   1. Rule-based: chatbots that answer questions based on some predefined rules on which it is trained on. 2. Artificial intelligence-based: chatbots that use some machine learning, statistical approaches, and natural language processing to understand the context and intent of a question and to respond to it. |
| Dialogue initiative | Who leads the conversation: user, chatbot, both? |
| Input modality | How the user interacts with the chatbot: spoken language, written language, visual language (facial expression and body language). |
| Output modality | How the chatbot interacts with the user: spoken language, written language, visual language (facial expression and body language). |
| Embodiment | Whether there is a virtual representation (e.g. avatar or virtual human) shown on the screen |
| Targeted disorder | The disorder that the chatbot was designed for. |
| **Outcome characteristics** |  |
| Measured outcome | What is the outcome that the study measured?  Attitude, acceptability, usability, satisfaction,… |
| Outcome measure | How was the outcome measured |
| **Findings** |  |
| Results of the study | The main findings for relevant outcomes |
