# Supplementary material for: Perceptions and Opinions of Patients About Mental Health Chatbots: Scoping Review
Source: J Med Internet Res. 2021 Jan 13;23(1):e17828. doi: 10.2196/17828 (PMC7840290; doi:10.2196/17828)
Supplement: Multimedia Appendix 3 [file jmir_v23i1e17828_app3.docx]

**Multimedia Appendix 3.** The metadata and population characteristics of each included study.

| Author (year)^ID^ | Country | Publication type | Study design | Sample size | Mean age | Sex (male) | Sample type | Recruitment Setting |
| --- | --- | --- | --- | --- | --- | --- | --- | --- |
| Ali (2018)^33^ | USA | Conference proc. | Survey | 9 | - | - | Clinical | Clinical |
| Auriacombe (2018)^68^ | France | Journal | Survey | 139 | 43 | 49% | Clinical | Clinical |
| Bickmore (2010)^53^ | USA | Journal | Survey | 131 | 48 | 54% | Clinical | Clinical |
| Bickmore (2010)^54^ | USA | Journal | Survey | 20 | - | 33% | Clinical | Clinical |
| Bresó (2016)34 | Spain & Mexico | Journal | Survey | 60 | - | 55% | Non-clinical | Educational |
| Burton (2016)^35^ | Romania, Spain, Scotland | Journal | Survey | 27 | 39 | 33% | Clinical | Clinical |
| Cameron (2018) ^36^ | UK | Conference proc. | Survey | 7 | - | 29% | Non-clinical | Clinical |
| Demirci (2018)^55^ | Turkey | Dissertation | Survey | 16 | 28 | 56% | Non-clinical | Educational |
| DeVault (2014)^56^ | USA | Conference proc. | Quasi-experiment | 351 | 42.6 | 62% | Non-clinical | Community |
| Elmasri (2016)^57^ | Australia | Conference proc. | Survey | 17 | - | 58.8% | Non-clinical | Educational |
| Fitzpatrick (2017)^37^ | USA | Journal | Survey | 70 | 22.2 | 33% | Clinical | Clinical |
| Fulmer (2018)^38^ | USA | Journal | Survey | 75 | 22.9 | 30% | Non-clinical | Educational |
| Grolleman (2006)^39^ | Netherlands | Conference proc. | Survey | 35 | - | - | Clinical | Educational |
| Inkster (2018)^40^ | Global population | Journal | Survey | 129 | - | - | Non-clinical | Community |
| Ku (2007)^41^ | Korea | Journal | Survey | 10 | 28.8 | 50% | Clinical | Clinical |
| Lahiri (2011)^65^ | USA | Journal | Survey | 8 | 16 | - | Clinical | - |
| Lisetti (2013)^58^ | USA | Journal | Quasi-experiment | 81 | - | - | Non-clinical | Educational |
| Luerssen (2018)^62^ | Australia | Conference proc. | Survey | 163 | 32 | - | Clinical | Community |
| Ly (2017)^42^ | Sweden | Journal | Survey | 28 | 26.2 | 46.4% | Non-clinical | Educational & Community |
| Martínez-Miranda (2014)^64^ | Spain | Conference proc. | Survey | 8 | - | 40% | Clinical | Community |
| Morries (2018)^67^ | USA | Journal | Survey | 1284 | 18.2 | 35% | Non-clinical | Community |
| Milne (2010)^59^ | Australia | Conference proc. | Survey | 14 | 10 | - | Clinical | - |
| Philip (2017)^66^ | France | Journal | Survey | 187 | 46.5 | 42.5% | Clinical | Clinical |
| Pinto (2015)^43^ | USA | Journal | Survey | 28 | 22 | 33% | Clinical | Community |
| Pontier (2008)^44^ | Germany | Conference proc. | Survey | 28 | - | - | Non-clinical | Community |
| Razavi (2016)^45^ | USA | Conference proc. | Survey | 5 | - | 80% | Clinical | - |
| Schroeder (2018)^46^ | USA | Conference proc. | Survey | 73 | 37.3 | 9.5% | Clinical | Clinical |
| Smith (2014)^47^ | USA | Journal | Survey | 37 | 48.2 | 48.6% | Clinical | Clinical |
| Smith (2014)^48^ | USA | Journal | Survey | 26 | 24.2 | 77% | Clinical | Clinical, Educational & Community |
| Smith (2015)^49^ | USA | Journal | Survey | 33 | 51 | 97% | Clinical | Clinical |
| Swartout (2013)^63^ | USA | Journal | Survey | 111 | 41 | 79.3% | Non-clinical | Community |
| Tanaka (2015)^50^ | Japan | Conference proc. | Survey | 30 | - | 73.3% | Non-clinical | Educational |
| Tielman (2017)^51^ | Netherlands | Journal | Survey | 4 | - | 50% | Clinical | Clinical |
| Tielman (2017)^52^ | Netherlands | Journal | Survey | 46 | 22.9 | 82.6% | Non-clinical | Educational |
| Wargnier (2018)^60^ | France | Journal | Survey | 14 | 78.8 | 21.4% | Clinical | Clinical |
| Yasavur (2014)^61^ | USA | Journal | Survey | 89 | - | 69.7% | Non-clinical | Educational |
| Yokotani (2018)^69^ | Japan | Journal | RCT | 55 | 22.9 | 45.5% | non-clinical | Educational |

**References**

33. Ali MR, Rasazi Z, Mamun AA, Langevin R, Rawassizadeh R, Schubert L, et al. A Virtual Conversational Agent for Teens with Autism: Experimental Results and Design Lessons. arXiv preprint arXiv:181103046. 2018.

34. Breso A, Martinez-Miranda J, Botella C, Banos R, Garcia-Gomez J. Usability and acceptability assessment of an empathic virtual agent to prevent major depression. Expert Systems: International Journal of Knowledge Engineering and Neural Networks. 2016 Aug;33(4):297-312. PMID: 2016-45132-001.

35. Burton C, Tatar AS, McKinstry B, Matheson C, Matu S, Moldovan R, et al. Pilot randomised controlled trial of Help4Mood, an embodied virtual agent-based system to support treatment of depression. Journal of Telemedicine and Telecare. 2016 Sep;22(6):348-55. PMID: 2016-40249-004.

36. Cameron G, Cameron D, Megaw G, Bond R, Mulvenna M, O’Neill S, et al., editors. Assessing the Usability of a Chatbot for Mental Health Care. Internet Science; 2019 2019//; Cham: Springer International Publishing.

37. Fitzpatrick KK, Darcy A, Vierhile M. Delivering Cognitive Behavior Therapy to Young Adults With Symptoms of Depression and Anxiety Using a Fully Automated Conversational Agent (Woebot): A Randomized Controlled Trial. JMIR Ment Health. 2017 Jun 6;4(2):e19. PMID: 28588005. doi: 10.2196/mental.7785.

38. Fulmer R, Joerin A, Gentile B, Lakerink L, Rauws M. Using Psychological Artificial Intelligence (Tess) to Relieve Symptoms of Depression and Anxiety: Randomized Controlled Trial. JMIR Ment Health. 2018 Dec 13;5(4):e64. PMID: 30545815. doi: 10.2196/mental.9782.

39. Grolleman J, van Dijk B, Nijholt A, van Emst A, editors. Break the habit! designing an e-therapy intervention using a virtual coach in aid of smoking cessation. International Conference on Persuasive Technology; 2006: Springer.

40. Inkster B, Sarda S, Subramanian V. An Empathy-Driven, Conversational Artificial Intelligence Agent (Wysa) for Digital Mental Well-Being: Real-World Data Evaluation Mixed-Methods Study. JMIR Mhealth Uhealth. 2018 Nov 23;6(11):e12106. PMID: 30470676. doi: 10.2196/12106.

41. Ku J, Han K, Lee HR, Jang HJ, Kim KU, Park SH, et al. VR-based conversation training program for patients with schizophrenia: a preliminary clinical trial. Cyberpsychol Behav. 2007 Aug;10(4):567-74. PMID: 17711366. doi: 10.1089/cpb.2007.9989.

42. Ly KH, Ly AM, Andersson G. A fully automated conversational agent for promoting mental well-being: A pilot RCT using mixed methods. Internet Interventions. 2017;10:39-46. doi: <http://0-dx.doi.org.wam.leeds.ac.uk/10.1016/j.invent.2017.10.002>.

43. Pinto MD, Greenblatt AM, Hickman RL, Rice HM, Thomas TL, Clochesy JM. Assessing the critical parameters of eSMART-MH: A promising avatar-based digital therapeutic intervention to reduce depressive symptoms. Perspectives in Psychiatric Care. 2015 Jul;52(3):157-68. PMID: 2016-32710-003.

44. Pontier M, Siddiqui GF. A Virtual Therapist That Responds Empathically to Your Answers. Intelligent Virtual Agents2008. p. 417-25.

45. Razavi SZ, Ali MR, Smith TH, Schubert LK, Hoque ME, editors. The LISSA virtual human and ASD teens: An overview of initial experiments. International Conference on Intelligent Virtual Agents; 2016: Springer.

46. Schroeder J, Wilkes C, Rowan K, Toledo A, Paradiso A, Czerwinski M, et al. Pocket Skills: A Conversational Mobile Web App To Support Dialectical Behavioral Therapy. Proceedings of the 2018 CHI Conference on Human Factors in Computing Systems; Montreal QC, Canada: ACM; 2018.

47. Smith MJ, Ginger EJ, Wright M, Wright K, Humm LB, Olsen D, et al. Virtual reality job interview training for individuals with psychiatric disabilities. Journal of Nervous and Mental Disease. 2014 Sep;202(9):659-67. PMID: 2014-37314-006.

48. Smith MJ, Ginger EJ, Wright K, Wright MA, Taylor JL, Humm LB, et al. Virtual reality job interview training in adults with autism spectrum disorder. Journal of Autism and Developmental Disorders. 2014 Oct;44(10):2450-63. PMID: 2014-19106-001.

49. Smith MJ, Humm LB, Fleming MF, Jordan N, Wright MA, Ginger EJ, et al. Virtual Reality Job Interview Training for Veterans with Posttraumatic Stress Disorder. J Vocat Rehabil. 2015;42(3):271-9. PMID: 27721645. doi: 10.3233/jvr-150748.

50. Tanaka H, Sakti S, Neubig G, Toda T, Negoro H, Iwasaka H, et al. Automated Social Skills Trainer. Proceedings of the 20th International Conference on Intelligent User Interfaces - IUI '152015.

51. Tielman ML, Neerincx MA, Bidarra R, Kybartas B, Brinkman WP. A Therapy System for Post-Traumatic Stress Disorder Using a Virtual Agent and Virtual Storytelling to Reconstruct Traumatic Memories. Journal of Medical Systems. 2017;41(8):125. PMID: 28699083.

52. Tielman ML, Neerincx MA, van Meggelen M, Franken I, Brinkman WP. How should a virtual agent present psychoeducation? Influence of verbal and textual presentation on adherence. Technology & Health Care. 2017;25(6):1081-96. PMID: 28800346.

53. Bickmore TW, Mitchell SE, Jack BW, Paasche-Orlow MK, Pfeifer LM, O'Donnell J. Response to a relational agent by hospital patients with depressive symptoms. Interacting with Computers. 2010 Jul;22(4):289-98. PMID: 2010-10513-007.

54. Bickmore TW, Puskar K, Schlenk EA, Pfeifer LM, Sereika SM. Maintaining reality: Relational agents for antipsychotic medication adherence. Interacting with Computers. 2010 Jul;22(4):276-88. PMID: 2010-10513-006.

55. Demirci HM. User experience over time with conversational agents case study of woebot on supporting subjective well-being: Middle East Technical University; 2018.

56. DeVault D, Artstein R, Benn G, Dey T, Fast E, Gainer A, et al. SimSensei kiosk: a virtual human interviewer for healthcare decision support. Proceedings of the 2014 international conference on Autonomous agents and multi-agent systems; Paris, France: International Foundation for Autonomous Agents and Multiagent Systems; 2014.

57. Elmasri D, Maeder A, editors. A Conversational Agent for an Online Mental Health Intervention. 2016; Cham: Springer International Publishing.

58. Lisetti C, Amini R, Yasavur U, Rishe N. I Can Help You Change! An Empathic Virtual Agent Delivers Behavior Change Health Interventions. ACM Transactions on Management Information Systems. 2013;4(4):1-28. doi: 10.1145/2544103.

59. Milne M, Luerssen MH, Lewis TW, Leibbrandt RE, Powers DMW, editors. Development of a virtual agent based social tutor for children with autism spectrum disorders. The 2010 International Joint Conference on Neural Networks (IJCNN); 2010 18-23 July 2010.

60. Wargnier P, Benveniste S, Jouvelot P, Rigaud AS. Usability assessment of interaction management support in LOUISE, an ECA-based user interface for elders with cognitive impairment. Technology and Disability. 2018;30(3):105-26. doi: http://0-dx.doi.org.wam.leeds.ac.uk/10.3233/TAD-180189.

61. Yasavur U, Lisetti C, Rishe N. Let’s talk! speaking virtual counselor offers you a brief intervention. Journal on Multimodal User Interfaces. 2014;8(4):381-98. doi: 10.1007/s12193-014-0169-9.

62. Luerssen MH, Hawke T. Virtual Agents as a Service: Applications in Healthcare. Proceedings of the 18th International Conference on Intelligent Virtual Agents; Sydney, NSW, Australia: ACM; 2018.

63. Swartout W, Artstein R, Forbell E, Foutz S, Lane HC, Lange B, et al. Virtual Humans for Learning. AI Magazine. 2013;34(4). doi: 10.1609/aimag.v34i4.2487.

64. Martínez-Miranda J, Bresó A, García-Gómez JM, editors. Look on the bright side: a model of cognitive change in virtual agents. International Conference on Intelligent Virtual Agents; 2014: Springer.

65. Lahiri U, Bekele E, Dohrmann E, Warren Z, Sarkar N. Design of a virtual reality based adaptive response technology for children with autism. IEEE Trans Neural Syst Rehabil Eng. 2013 Jan;21(1):55-64. PMID: 23033333. doi: 10.1109/tnsre.2012.2218618.

66. Philip P, Micoulaud-Franchi JA, Sagaspe P, Sevin E, Olive J, Bioulac S, et al. Virtual human as a new diagnostic tool, a proof of concept study in the field of major depressive disorders. Scientific Reports. 2017;7:42656. PMID: 28205601.

67. Morris RR, Kouddous K, Kshirsagar R, Schueller SM. Towards an Artificially Empathic Conversational Agent for Mental Health Applications: System Design and User Perceptions. J Med Internet Res. 2018;20(6):e10148. PMID: 29945856. doi: 10.2196/10148.

68. Auriacombe M, Moriceau S, Serre F, Denis C, Micoulaud-Franchi J-A, de Sevin E, et al. Development and validation of a virtual agent to screen tobacco and alcohol use disorders. Drug and Alcohol Dependence. 2018 Dec;193:1-6. PMID: 2018-59342-002.

69. Yokotani K, Takagi G, Wakashima K. Advantages of virtual agents over clinical psychologists during comprehensive mental health interviews using a mixed methods design. Computers in Human Behavior. 2018 Aug;85:135-45. PMID: 2018-24910-015.
